# A randomised controlled trial of caseload midwifery care: M@NGO (Midwives @ New Group practice Options)

**DOI:** 10.1186/1471-2393-11-82

**Published:** 2011-10-26

**Authors:** Sally K Tracy, Donna Hartz, Bev Hall, Jyai Allen, Amanda Forti, Anne Lainchbury, Jan White, Alec Welsh, Mark Tracy, Sue Kildea

**Affiliations:** 1 Midwifery and Women's Health Research Unit, Royal Hospital for Women, Barker Street, Randwick, New South Wales, 2031; 2 Australian Catholic University & Mater Medical Research Institute, Level 1 Aubigny Place, Raymond Terrace, South Brisbane, Queensland, 4101 Australia; 3 Royal Hospital for Women, Barker Street, Randwick, New South Wales, 2031 Australia; 4 Department of Maternal Fetal Medicine, University of New South Wales, Randwick, New South Wales, 2031 Australia; 5 Centre for Newborn Care, Westmead Hospital, Cnr Hawkesbury & Darcy Roads, Westmead, New South Wales, 2145 Australia; 6 University of Sydney, Sydney, New South Wales, 2006 Australia

## Abstract

**Background:**

Australia has an enviable record of safety for women in childbirth. There is nevertheless growing concern at the increasing level of intervention and consequent morbidity amongst childbearing women. Not only do interventions impact on the cost of services, they carry with them the potential for serious morbidities for mother and infant.

Models of midwifery have proliferated in an attempt to offer women less fragmented hospital care. One of these models that is gaining widespread consumer, disciplinary and political support is caseload midwifery care. Caseload midwives manage the care of approximately 35-40 a year within a small Midwifery Group Practice (usually 4-6 midwives who plan their on call and leave within the Group Practice.) We propose to compare the outcomes and costs of caseload midwifery care compared to standard or routine hospital care through a randomised controlled trial.

**Methods/design:**

A two-arm RCT design will be used. Women will be recruited from tertiary women's hospitals in Sydney and Brisbane, Australia. Women allocated to the caseload intervention will receive care from a named caseload midwife within a Midwifery Group Practice. Control women will be allocated to standard or routine hospital care. Women allocated to standard care will receive their care from hospital rostered midwives, public hospital obstetric care and community based general medical practitioner care. All midwives will collaborate with obstetricians and other health professionals as necessary according to the woman's needs.

**Discussion:**

Data will be collected at recruitment, 36 weeks antenatally, six weeks and six months postpartum by web based or postal survey. With 750 women or more in each of the intervention and control arms the study is powered (based on 80% power; alpha 0.05) to detect a difference in caesarean section rates of 29.4 to 22.9%; instrumental birth rates from 11.0% to 6.8%; and rates of admission to neonatal intensive care of all neonates from 9.9% to 5.8% (requires 721 in each arm). The study is not powered to detect infant or maternal mortality, however all deaths will be reported. Other significant findings will be reported, including a comprehensive process and economic evaluation.

**Trial registration:**

Australian New Zealand Clinical Trials Registry ACTRN12609000349246

## Background

Australia has an enviable record of safety for women in childbirth [1]. There is nevertheless growing concern at the increasing level of intervention, cost and consequent morbidity amongst childbearing women [2]. Rising intervention rates are most clearly reflected in the changes in rates of caesarean section which have increased over time. In 1991, only 19.0% of births were via caesarean section. By 2006, 31% of all births were via caesarean [1]. In addition to caesarean section, population based research in Australia revealed that amongst women with uncomplicated pregnancies, a third of all women currently have some form of intervention such as an induction or augmentation of their labour combined with an epidural [3]. Cost modelling of these interventions showed a relative cost increase of up to 50% for low risk primiparous women and up to 36% for low risk multiparous women as labour interventions accumulated [4].

Not only do interventions impact on the cost of services, they carry with them the potential for serious morbidities for mother and infant. Over a decade ago a large population based study in Victoria found that more than nine out of ten women had at least one significant medical complaint after giving birth [5]. Compared with spontaneous vaginal births, women having an instrumental birth had increased odds for perineal pain (OR 4.69, 95%CI 3.2-6.8) sexual problems (OR 2.06, 95% CI 1.4-3.0), and urinary incontinence (OR 1.81, 95% CI 1.1-2.91) [5]. Other studies have shown that a first birth by forceps delivery can cause a twofold increase in the risk of having persistent faecal incontinence, and in about half these cases faecal incontinence may persist for at least five years [6]. Caesarean births carry the risk of increased perinatal mortality in subsequent pregnancies[1,7,8] and the risk of morbidity associated with surgery[9] and possible maternal complications in subsequent pregnancies (e.g., uterine rupture, placenta praevia, and placenta accreta) [10,11]. Operative birth also increases the fetal risks of respiratory distress syndrome [12] persistent pulmonary hypertension,[13] and admission to special care or neonatal intensive care nurseries particularly if the caesarean section is performed before the onset of labour [12,14,15]. Australian research into the rates of admission to neonatal intensive care of term babies of low risk women found the overall rate of admission is currently as high as 7.32% amongst low risk women [15].

An intervention that could lower rates of caesarean section, instrumental birth and epidural analgesia with no increase in mortality or morbidity for the mother or baby would improve the quality of maternity care, reduce unnecessary or harmful interventions and be cost effective.

No intervention reported in the Cochrane Library of systematic reviews of pregnancy and childbirth has had a more significant effect on lowering rates of intervention during childbirth than 'continuity of care' [16]. However, in Australia at present fewer than10% of women have access to continuity of care [17] and some women may meet up to 20 different midwives or caregivers from the time they begin antenatal care until the baby is born and the mother is discharged from postnatal care. Models of midwifery have proliferated in an attempt to offer women more continuity of care. We proposed to compare the outcomes and costs of caseload midwifery care compared to standard or routine hospital care for childbearing women through a randomised controlled trial.

### Caseload midwifery

The aim of caseload midwifery is to provide women with the same midwife (or small group practice of midwives) to look after them from booking in through until the time they are discharged from care at about four to six weeks following the birth of the baby.

Despite the substantial health care costs associated with maternal and infant health, there are minimal data on the cost effectiveness of maternity care offered in the public (or private sector) in Australia. Allocating finite resources in ways that are most effective in improving health outcomes continues to be a challenge [18]. As yet there is no cost benefit analysis of the caseload midwifery model for women of all risk anywhere in the world.

There remains continuing uncertainty and debate about the risks, benefits and costs of midwifery led models versus other models of maternity care [16,19,20]. Several large national reviews [17,21] and a workforce study [22] identified that the current limited and inflexible scope of practice negatively affects Australian midwives' sense of satisfaction with their employment. The Commonwealth Health Workforce Review [23] found that workforce shortages, inflexibilities and inefficiencies in workplace arrangements are major contributors to poor health outcomes.

We plan to implement and evaluate Midwifery Group Practices at two large teaching maternity hospitals in Australia to compare the outcomes and costs of caseload midwifery care and standard or routine hospital care for childbearing women.

Ethical approval and site specific ethical approval was received from all University and Area Health Service Human Research Ethics Committees governing the study sites: University of Sydney HREC approval REF NO 12068; Lead HREC approval REF NO 0805072M.

## Methods/design

The study uses a pragmatic two arm, unblinded randomised controlled design, to compare caseload midwifery care with standard maternity care.

### Aims and Objectives

The aim of the study is to compare the outcomes and costs of caseload midwifery care compared to standard or routine hospital care for childbearing women through a randomised controlled trial.

**Primary objectives **are to determine whether women receiving caseload midwifery care experience the same rates of caesarean section, instrumental births and use of epidural analgesia compared to women receiving routine care and to determine whether women with identified risk factors at the onset of labour experience the same rates of neonatal morbidity (including admission to the neonatal intensive care unit), and perinatal mortality compared to routine care.

We will determine whether caseload midwifery care costs the same as routine maternity care and whether women who receive caseload care experience the same potential benefits and consequences of postnatal care as women receiving routine maternity care.

**Secondary objectives **are to determine whether midwives offering caseload practice experience the same levels of work satisfaction and the same levels of productivity as midwives working in conventional rostered and rotating shifts; and to determine whether obstetricians working with caseload midwives experience the same levels of professional satisfaction as obstetricians working with rostered midwives in the routine work setting. We will also determine the rates of postnatal depression and smoking in pregnancy.

### Outcome measures

The *primary maternal outcomes *of the study are the proportion of women in each arm of the study:

• having caesarean section operations,

• instrumental births

• spontaneous vaginal births

• epidural analgesia for labour and birth

The *primary neonatal outcomes *of the study are the proportion of infants in each arm of the study who have:

• Apgar scores less than or equal to 7 at 5 minutes

• preterm birth (less than 37 completed weeks)

• admission to special care nursery or neonatal intensive care unit;

*Secondary maternal outcomes *include the proportion of women in each arm of the study who have:

• antenatal admissions

• induction and/or augmentation of labour

• women who are breastfeeding at six weeks and six months

*Cost outcomes *include:

• Comparative average cost per mother and baby episode as an AR-DRG

In addition to the primary outcomes listed above we will report on

*Other neonatal outcomes *including: low birth weight (less than 2500 g); length of neonatal hospital stay; neonatal convulsions; neonatal trauma (fracture or palsies); neonatal resuscitation: use of neonatal respiratory support (mechanical ventilation/CPAP) and/or perinatal mortality.

*Other maternal outcomes *include the rate of maternal complications (e.g. hypertension, ante partum haemorrhage, cord prolapse etc) and miscarriages in both the intervention and control arms; the proportion of women who have perineal trauma (episiotomy, 1st, 2nd, 3rd or 4th degree perineal tear, sutures required); postpartum haemorrhage (more than 500 ml or any amount which causes deterioration in maternal health; and/or blood transfusion); and serious maternal complications (e.g. intensive care unit admission or death).

*Although the study is not powered to detect other related outcomes including measures of perinatal mortality (e.g. fetal or neonatal death,) or maternal mortality, these events will be reported in full*.

### Study population

Pregnant women booking in to give birth at one of two sites during the recruitment period will be invited to participate in the study and will be randomly allocated to caseload midwifery care versus routine care according to a post-consent method using accepted concealment measures endorsed by the NHMRC. The two sites are - large tertiary maternity hospitals in Sydney (site 1) and Brisbane (site 2).

### Study Power

With 750 women or more in each of the intervention and control arms the study is powered (based on 80% power; alpha 0.05) to detect a difference in caesarean section rates of 29.4 to 22.9%; instrumental birth rates from 11.0% to 6.8%; and rates of admission to neonatal intensive care of all neonates from 9.9% to 5.8% (requires 721 in each arm). The study is not powered to detect infant or maternal mortality, however all deaths will be reported. Secondary outcomes will include rates of smoking cessation (eg 14.8% to 9.9% needing 747 women in each arm) and breastfeeding initiation and duration. We have calculated a sample to include a 30% attrition rate; therefore we aim to recruit a total of 1,950 women. All outcome measures identified by the Cochrane Protocol [19] will be collected. Individual hospital data bases and a purposefully designed data base will collect all primary and secondary outcome and process measures in addition to most cost outcome measures.

#### Definitions of Control and Intervention

The lead professional for the continuum of the antenatal, intrapartum and postnatal periods will be the criterion for the classification of care as caseload midwifery or routine (medical doctor-led) care [19].

***CONTROL **Standard or Routine care*: the designated obstetrician is the lead professional. Women are booked under the hospital consultant on call for the day of booking, and the responsibility for the delivery of care, from initial booking through the postnatal period, rests with the public maternity hospital service. Women allocated to the control group can choose from the standard hospital options for care which include midwives clinic antenatally; GP shared care antenatally; followed by general public hospital care in labour and in the postnatal ward. This may involve women seeing a different midwife for every visit; care by junior medical obstetric staff; or shared care with an accredited general medical practitioner (GP) (i.e. the GP provides the woman's antenatal care, usually nearer to her home, but the woman is booked for extra antenatal care, labour, birth and postnatal care at the hospital). Women may see an obstetrician during pregnancy with other referrals or consultation as necessary. When women come into the hospital for labour, birth and postnatal care they will be cared for by whichever midwives and doctors are rostered for duty. For women in the control and intervention arms at each site the care will be provided according to the same hospital guidelines and protocols of each site.

***INTERVENTION **Caseload Midwifery care: *Women allocated to the caseload intervention will receive antenatal, intrapartum and postpartum care from a named caseload midwife who will be backed up when necessary by other caseload midwives from within her Midwifery Group Practice. Each named caseload midwife works in a Midwifery Group Practice of four full time equivalent midwives each working a cycle of 152 hours during each four week period. Caseload midwives must be independent of the hospital rosters and employed on an annual salary to allow for workload to be self managed within each Midwifery Group Practice and responding to the needs of the women in their caseload. The named midwife is on call for the woman's labour and birth except in designated circumstances such as annual leave; sick leave; having more than one woman in labour; or if it is on one of the two days per week that the named midwife is scheduled to not work or be on call. Care will then be provided by a back-up caseload midwife from the Midwifery Group Practice. The caseload midwife is the woman's lead professional but one or more consultations with medical doctors may be part of routine practice [24]. Midwifery care is offered simultaneously with medical care if required. In addition to providing care until after the birth of the baby, the named caseload midwife (or a back up midwife from the Midwifery Group Practice) will attend the hospital on most days to provide some postnatal care until discharge and will provide full domiciliary care following discharge from hospital. Care will be provided according to hospital guidelines and protocols for up to six weeks following birth.

#### Trial Eligibility

Women will be eligible for trial entry if they are less than 24 completed weeks of pregnancy and a minimum of 18 years old at booking in. At the participating centres research midwives will recruit the required number of women in the experimental arm over 24 months. We will enroll 1950 women in anticipation of no more than a 30% attrition rate.

#### Exclusions

Women will be excluded from the trial if they are electively booked to give birth via caesarean section at the time of booking in; or are already booked with a named care provider (Obstetrician/GP/midwife).

#### Trial Recruitment

At site [1] women will be recruited using three strategies depending on point of first contact.

**1**. When the woman contacts the hospital by phone for booking, the administration desk will send out information leaflets informing women of (a) the opportunity to book with a caseload midwife as part the care offered at the hospital and (b) inviting them to participate in the study before the first visit to the hospital antenatal clinics. Women will attend the hospital antenatal clinic for their booking visit. At the first visit (booking clinic) women will be seen by a research midwife who ascertains if the woman has previously received written information and if so, she will be invited to participate. Following written consent, the midwife will randomise the woman to the intervention caseload midwifery care with a named midwife or routine care via a central telephone randomisation service (NH&MRC method); and enter details on the Trial Register and Daily Log Book. (The NHMRC administered telephone randomisation method guarantees both random sequence generation and allocation concealment according to CONSORT guidelines).

**2**. When a booking referral from the woman's General Practitioner is sent to the hospital, the administration desk contacts the woman and informs the woman of (a) the opportunity to book with a caseload midwife as part the care offered at the hospital, and (b) if eligible invite them to participate in the study before the first booking visit. Interested women will be sent information leaflets. The woman is contacted by phone by the research midwife to ensure the information has been received, assess the woman's understanding of the study, answer any questions, and obtain verbal consent to participate and be randomised. Once verbal consent is obtained the trial procedure is the same as that above and her details are entered onto the Trial Register and Daily Log Book. Consent is formalised in writing at the first booking visit. At this stage, if the woman declines to give written consent or is ineligible, she is not enrolled in the trial. Women not wishing to participate in the study will progress through the antenatal clinic as usual.

**3**. When the woman has not received written information, at the booking clinic, she will be seen by a research midwife, who will provide her with written information and an opportunity to discuss the study. Before being invited to participate in the study, the woman will be given the opportunity to defer her decision to participate until the next antenatal appointment where she will be seen again by the antenatal clinic midwives and the research midwife. If the woman wishes to decline or accept the invitation to participate at this first visit, she may do so. She may contact the research midwives at a later time to participate in the study. Once women have given written consent to be involved in the trial, the trial procedure is the same as that above. Details of these women will also be recorded in the Trial Register and Daily Log Book Women not wishing to participate in the study will progress through the antenatal clinic as usual.

**At Site 2 **pregnant women will be randomly allocated to caseload midwifery care or routine care during pregnancy.

1. On receipt of referral from the general practitioner, the GP Liaison will identify women eligible for caseload care. This process is guided by availability of places on caseload group practice models of care in association with the locality of the home address of women who book.

2. Women who qualify on these grounds will be telephoned to be informed of their acceptance to book at Site 2 and the availability of caseload midwifery care in their area. They will have the models of care briefly outlined, and be invited to participate in the trial.

3. Interested women will receive a brochure on models of care, and a M@NGO trial brochure in the post.

4. One week following postage, the research midwife will provide a follow up phone call to ensure the information was received, assess the woman's understanding of the study, answer any questions, and obtain verbal consent to participate and be randomised. We do not expect that this will create unnecessary delays as at present women may wait several weeks before they receive a letter confirming their acceptance to Site 2, with the models of care brochure and a booking appointment.

5. Once verbal consent is obtained and documented, the research midwife will randomise the woman to caseload or routine care via a central telephone randomisation service (NH&MRC method); and enter details onto the Trial Register and Daily Log Book

6. At the first booking visit women who were telephone randomised to take part in the study will confirm and formalise participation in the trial by giving written consent at the first booking visit in the home.

7. In the standard care model written consent will be obtained at the first booking visit in the hospital or community-based antenatal clinic.

8. At this stage, if the woman declines to given written consent or is ineligible (meets exclusion criteria) she is excluded from trial, and her care will be 'as usual' i.e. standard care.

Once women have given written consent to be involved in the trial, the trial procedure is the same as Site 1 above. Women not wishing to participate in the study will progress through the hospital or community antenatal clinics as usual. Details of these women will also be recorded in the Trial Register and Daily Log Book.

### Differences between Site 1 and Site 2

Although there is a slight difference in the recruitment process for the trial at the two sites, the integrity of the randomisation process is safeguarded by the fact that there is equipoise in the decision process for women regarding which model of care they will receive. Women who have a stated preference for either model are not invited to be randomised to a model of care. The proposed recruitment method for Site 2 differs slightly from the process employed at site 1 on two counts:

i. At site 1 all women attend a first booking visit at the hospital antenatal clinic before they are allocated to a model of care, including caseload care. At Site 2 women are allocated to a model of care, including caseload care by the GP Liaison, *prior to *the first booking visit.

ii. At site 1 the first booking visit occurs in the antenatal clinic, which enables research midwives' to provide information about the trial and obtain written consent. At Site 2 women are offered information by telephone and the initial consent is received by telephone. All women allocated to MGP have a home booking visit and do not attend the antenatal clinic at all. It is not feasible for the research midwife to accompany MGP midwives to every home visit to give information about the trial and obtain written consent in the first instance. See above the process for Site 2 to further clarify.

### Analysis

Analysis will be by intention to treat which will include withdrawals and losses to follow up. There should be minimal differences in the baseline characteristics between groups due to the randomisation process. Statistical adjustment may be needed if important differences arise in baseline characteristics. Relative risks with 95% confidence intervals for the primary outcomes will be calculated. Measures of categorical data will be analysed with chi-squared tests and continuous data will be analysed with t-tests. Logistic regression and multiple linear regressions will be used if necessary to adjust for confounding for binary and continuous outcomes.

Risk will be controlled for in the statistical analysis and identified at the onset of labour rather than at enrolment in the study. Women will be categorised dichotomously as 'at low risk' and 'not low risk' according to maternal risk status at the time of the onset of labour. The risk factors and levels of risk are well defined in the ACM Guidelines [24] as level B or C - however, all definitions of risk will be available in the publication of the study.

#### Interim Analysis

A data monitoring group will look for differences between the groups that may be larger than expected as well as unanticipated adverse effects that may occur [25]. After 50% of the women have given birth a difference of at least three standard deviations in interim analysis of a major endpoint is needed to justify stopping the trial. (All perinatal deaths will be reviewed by a multidisciplinary adverse events committee blinded to treatment allocation.) While it is not possible to blind participants to the model of care they receive, we will endeavour to blind the outcome assessments.

#### Cost methods

As no single outcome measure can encapsulate all benefits of treatment, the economic evaluation is based on a cost-consequence analysis [26]. We will determine the importance of each outcome measure relative to the costs if the caseload midwifery care and routine care costs are similar. The costs will be presented in terms of average cost per mother and baby and reported as a 'mother/baby episode' which includes the full episode of care. Costs will also be presented in actual Australian Revised Diagnosis Related Group (ARDRG) related funding terms for each arm of the study.

Expenditure data will be obtained from each hospital's financial system including detailed patient-level data on inpatient contacts for the mother and baby and the data related to the costing of medical and midwifery contact time during each mother/baby episode.

### Qualitative methods

#### Women's Questionnaires

Women will be asked to assess the service through qualitative surveys offered at thirty six weeks, six weeks and six months postpartum. At six weeks women will be offered a survey tool based on the Oakley et al 'social support' questionnaire [27]. This questionnaire is validated for use amongst women considered to be both high and low risk as well as those of lower socio-economic status. At six months women will be offered the 36 item short form SF36 [28] which will provide subjective accounts of health following childbirth. We have chosen these tools rather than the conventional satisfaction type surveys because satisfaction may reflect whether or not expectations have been met rather than whether or not benefit has been achieved in the eyes of the woman and her family.

#### Questionnaires for Staff

The team plans to collaborate with researchers from the department of Organisation and Management at the Australian School of Business based at UNSW to undertake a rigorous assessment of employees' motivation, well-being and emotion management strategies, as well as the drivers and outcomes of these factors particularly in relation to measuring the effect of a new model of delivering midwifery care, compared to traditional models of delivering midwifery care.

### Confidentiality and Data Security

Participants in the trial will be identified by a study number only, with a master code sheet linking names with numbers being held securely and separately from the study data. See ethics section for storage and disposal details.

### Group Allocation

The randomisation schedule will be prepared by a researcher not involved with treatment allocation. Following written informed consent from the woman, the research midwife will telephone the randomisation service. Details of the woman's consent and trial entry data will be recorded. The midwife will then be informed of the group (caseload midwifery or routine hospital care) to which the woman has been randomly allocated and the woman will be issued with a unique 4 digit study number. Study group allocation will be recorded on the Trial Register and Daily Log Book. To assess the comparability of the study groups, baseline demographic and medical information will be collected from the medical record at the time of entry into the study. Because the structure of practice is so different; there should be minimal opportunity for cross overs to occur. No attempt will be made to blind the identification of women randomised to either arm of the study.

#### Justification for RCT

The strength of the proposed experimental randomised design is the capacity to determine causality between the outcome variables (dependent variables) and the type of care received by women in each of the two groups (independent variables).

In a random assignment to each nominal group: the probability of being assigned to intervention or control is not dependent on pre-trial choice or preference and any other baseline patient characteristics. Women not allocated a caseload midwife will be cared for as usual in the routine hospital group by rostered midwives and rostered medical personnel. Some women may receive their antenatal care in a GP/shared care model and enter the hospital at the time of birth to be cared for by the rostered midwifery and medical staff on duty. Subjects will be analysed within the group to which they were allocated, irrespective of whether they experienced the intended intervention (intention to treat analysis). All exclusions will be reported.

## Discussion

This trial will provide a comprehensive and rigorous evaluation of caseload midwifery maternity care. The evidence that this trial can provide is long overdue for maternity policy makers and service providers who are responsible for the effective design, delivery and costs of services that are the most frequent cause of hospitalisation in Australia today.

Restructuring maternity services to introduce caseload midwifery care involves radical changes to conventional or routine midwifery and obstetric practices [29-31]. All these changes make an impact on health planning and the allocation of finite resources [18]. Many innovations are introduced in a relative policy vacuum. Models of maternity care are no exception. This proposed trial is significantly innovative because it is designed to be undertaken on as a multisite study in two different states within Australia and includes women of *all risk *rather than *low risk *women. Because our proposed trial meets the criteria of the Cochrane Systematic Review of midwifery caseload care in methods; randomisation; definitions of the intervention and control arms; outcome measures and statistical analysis,[19] the results will contribute to a wider inquiry and meta-analysis in the future.

In addition to evidence on the experiences of women receiving caseload midwifery care and the experiences of obstetricians and midwives offering caseload care, the outcomes of the proposed trial will contribute:

1. Level 1 evidence of the safety and effectiveness of having a known caseload midwife for the continuum of pregnancy birth and postnatally.

2. Level 1 evidence of the safety and effectiveness of caseload midwifery care for women of all risk.

3. Level 1 evidence on the cost effectiveness of caseload midwifery care and routine obstetric care for all women.

4. 'Australian randomised controlled trial data to the Cochrane systematic review of midwife led care[19].

## Competing interests

The authors declare that they have no competing interests.

## Authors' contributions

ST conceived of the study, participated in its design and coordination, drafted the manuscript and gave final approval of the version to be published. SK participated in the study design and coordination and helped draft the manuscript. DH, BH, AL, JA, AF participated in the design of the study, helped draft the manuscript and are managing the day to day conduct of the study. AW participated in the study design and coordination MT participated in the study design and coordination, helped to draft the manuscript and will perform the statistical analysis. JW is the overall manager of the midwifery group practices and participated in the study design and coordination. All authors read and approved the final manuscript.

## Pre-publication history

The pre-publication history for this paper can be accessed here:

http://www.biomedcentral.com/1471-2393/11/82/prepub
